# The secreted integrin ligand *nephronectin* is necessary for forelimb formation in *Xenopus tropicalis*

**DOI:** 10.1016/j.ydbio.2010.10.015

**Published:** 2011-01-15

**Authors:** Anita Abu-Daya, Satoko Nishimoto, Lynn Fairclough, Timothy J. Mohun, Malcolm P.O. Logan, Lyle B. Zimmerman

**Affiliations:** Division of Developmental Biology, MRC-National Institute for Medical Research, Mill Hill, London, NW7 1AA, UK

**Keywords:** *Xenopus tropicalis*, Genetics, Limb, Mutant, Nephronectin, Integrin

## Abstract

While limb regeneration has been extensively studied in amphibians, little is known about the initial events in limb formation in metamorphosing anurans. The small secreted integrin ligand *nephronectin* (*npnt*) is necessary for development of the metanephros in mouse. Although expressed in many tissues, its role in other developmental processes is not well-studied. Here we show that a transgene insertion that disrupts this gene ablates forelimb formation in *Xenopus tropicalis*. Our results suggest a novel role for integrin signalling in limb development, and represent the first insertional phenotype to be cloned in amphibians.

## Research Highlights

►The *Xenopus tropicalis* mutation *xdm* lacks all forelimb structures. *►Xdm* results from a transgene integration in the *nephronectin* gene. *►Xdm* tadpoles do not express *tbx5* in the prospective forelimb field. *►Xdm* is the first insertional mutation cloned in amphibians.

## Introduction

Limb formation is among the most intensively studied models for vertebrate organogenesis, incorporating initiation, growth, patterning of axes, regulated apoptosis, and specification and differentiation of specific cell types (Butterfield et al., 2010; Duboc and Logan, 2009; Logan, 2003). Amphibian limbs are also extensively used to understand regeneration (Beck et al., 2009; Brockes, 1997). Frog limb formation, however, is unique in vertebrate organogenesis. While all other organ systems (including limbs in urodele amphibians and other vertebrate taxa) form in parallel relatively early during embryogenesis, metamorphosing frog larvae develop limbs *de novo* after several weeks or more as free-swimming, growing tadpoles. In this respect, the prospective anuran limb has been compared to the insect imaginal disc, persisting in the larva in an undifferentiated state until specific cues are provided (Brown et al., 2005a), but the specific lineages and molecular signals responsible for limb initiation in these organisms remain unknown. Understanding how the metamorphosing tadpole initiates and patterns limbs that are highly homologous to those of other vertebrates in this unique context will elucidate processes critical for vertebrate limb formation and regeneration.

In amniotes, limb skeletal elements form as a result of outgrowth of flank lateral plate mesoderm cells (Capdevila and Izpisua Belmonte, 2001). The first molecular evidence for the specification of the forelimb-forming region is the expression of the T-box transcription factor, *Tbx5*. The requirement for *Tbx5* in forelimb formation is conserved in vertebrate models from zebrafish to mouse (Ahn et al., 2002). Conditional mouse mutants in *Tbx5* fail to initiate forelimb bud formation, and all of the elements of the forelimb appendicular skeleton, including the scapula and clavicle, fail to form (Rallis et al., 2003). Mutations in human TBX5 are associated with Holt–Oram syndrome, a dominant disorder characterised by forelimb deformities and heart defects (Basson et al., 1997; Li et al., 1997). Limb formation in urodele amphibians has been studied extensively using embryological methods (Stephens, 1999; Harrison, 1918). As in amniotes, limb initiation occurs in parallel with other organogenesis processes. At early stages, the potential for limb formation extends nearly the entire length of the lateral plate, then becomes gradually restricted to the anterior and posterior limb fields, with the intervening flank mesoderm playing a role in positioning and polarizing the forelimb (Slack, 1976, 1977).

Analysis of limb initiation in the free-swimming anuran larva has largely been limited to descriptive studies. The hindlimb bud consists of mesenchyme originating from a thickening of the somatopleure, which migrates from the coelomic epithelium to establish close contact with the thickened epidermis opposite (Balinsky, 1931; Filatow, 1933; Tschernoff, 1907). An external hindlimb bud is visible at stage 46 in *Xenopus*, with forelimb buds appearing subsequently at stage 48, in contrast to amniotes where forelimb formation precedes hindlimb. The molecular mechanism governing timing of limb bud outgrowth is not known. Unlike most processes in metamorphosis, which are controlled by the general regulator thyroid hormone (TH), initial formation of the limb bud occurs in the presence of the TH-blocking goitrogen methimazole (Brown et al., 2005a) although subsequent outgrowth is halted. As in amniotes, the position of the forelimb appears to coincide with the anterior limit of mesodermal *hoxc-6* expression (Burke et al., 1995), although *fgf8* is not expressed in the prospective limb prior to budding (Christen and Slack, 1997), and dorsoventral patterning also appears to use a different mechanism than in amniotes (Christen and Slack, 1998). Limb muscles likewise may have a separate origin. In other tetrapods, these derive from myogenic progenitors that migrate from the somites into the nascent limb bud (Christ and Brand-Saberi, 2002). When *Xenopus* muscle lineages were traced by transiently expressing Cre recombinase in somites of floxed RFP/GFP transgenic embryos, body wall and tail muscles were consistently permanently labelled. Interestingly, limb muscles in these animals were conspicuously never labelled, suggesting that frog limb myogenic precursor cells are not committed during somitogenesis at early larval stages and may derive from an alternative lineage (Satoh et al., 2005).

Nephronectin was first identified in screens for secreted ligands with the capacity to bind integrin α8β1 (Brandenberger et al., 2001; Morimura et al., 2001), and comprises five epidermal growth factor-like repeats, a linker segment containing the RGD cell adhesion motif, and a meprin-A5 protein receptor protein-tyrosine phosphatase *μ* (MAM) domain. Mouse knockouts of either *nephronectin* or its receptor show similar defects in epithelial–mesenchymal interactions in kidney organogenesis (Linton et al., 2007; Muller et al., 1997). The amphibian larval kidney (pronephros) abuts the forelimb-forming region, potentially playing a role in its formation. Intriguingly, extirpation studies in chick have implicated nephrogenic mesoderm in forelimb outgrowth (Geduspan and Solursh, 1992), although this model has subsequently been challenged (Fernandez-Teran et al., 1997; Perantoni et al., 2005). Here we report a transgene integration disrupting *nephronectin* in *Xenopus tropicalis*. The resulting *xenopus de milo* (*xdm*) phenotype lacks all detectable forelimb structures from the earliest stages of limb formation and abrogates *tbx5* expression in the prospective forelimb region. No other tissues, including hindlimb, appear to be affected. This mutation provides the first evidence for integrin signalling in limb initiation, and is also the first insertional mutation to be cloned in amphibians.

## Results

### *Xenopus de milo* forelimb mutation disrupts *nephronectin*

**Fig. 1:**
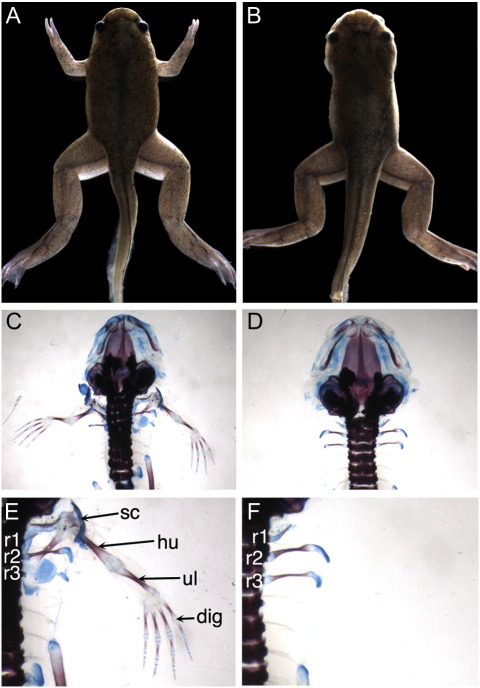
A transgene insertion ablates all forelimb structures. (A) Metamorphosing froglets heterozygous for an *Nkx2*.*5–*GFP transgene show normal forelimb development while (B) homozygous siblings lack forelimbs. (C, E) Skeletal preparation of a wild type froglet showing limb skeletal elements including the scapula (sc), humerus (hu), ulna (ul), and digits (dit); clavicle is hidden beneath the scapula. (D, F) All forelimb skeletal structures, including the scapula and clavicle, are missing in *xdm* froglets, but the ribs are unaffected (r1, r2, and r3).

**Fig. 2:**
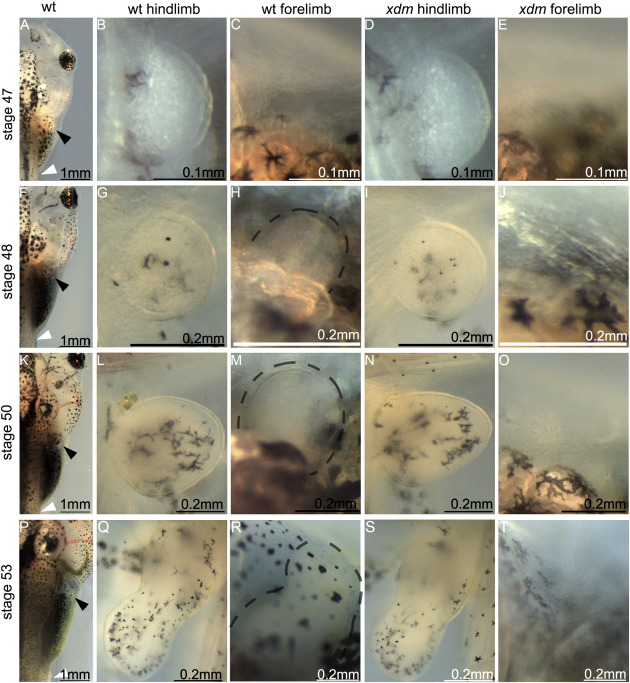
Forelimb buds do not form in *xdm*. (A, F, K, P) Dorsal views of stages 47, 48, 50 and 53 wt tadpoles showing sites of the forelimb (black arrowheads) and the hindlimb (white arrowheads). (D, I, N, S) Hindlimb development occurs normally in the mutant compared with the wt (B, G, L, Q). (C, E) Stage 47 wild type and mutant tadpoles immediately prior to forelimb bud initiation. (H) Forelimb buds (dashed outline) first appear at stage 48 in wt tadpoles but not in *xdm* homozygotes (J). (M) Forelimbs are more distinct in stage 50 wt larvae but are still absent in *xdm* (O). (R) By stage 53 the wt forelimb has formed a handplate, while mutant tadpoles still lack forelimb buds (T).

The *xenopus de milo* (*xdm*) mutation was identified in a transgenic line containing an 8 kb fragment of the *X*. *laevis Nkx2*.*5b* promoter (Sparrow et al., 2000) driving GFP in the developing heart. Offspring of sibling crosses develop normally to stage 47, forming wild type hindlimbs, but a roughly Mendelian fraction (43/211, 20%) had no forelimbs (Figs. 1A and B). *Xdm* skeletal preparations lack all elements of the forelimb appendicular skeleton including the shoulder girdle (scapula and clavicle, Figs. 1C–F). The axial skeleton and hindlimbs are unaffected. We then examined mutant embryos at earlier stages beginning immediately prior to forelimb bud initiation (stage 47) to stage 53 when a developing handplate is obvious. In *xdm* mutants, no forelimb structures, including the earliest limb bud, are detected at any stage analyzed (Figs. 2E, J, O, and T). The timing and morphology of hindlimb formation are unaffected in the mutant (Figs. 2D, I, N, and S). A separate transgenic line bearing an independent insertion of the same construct showed no defects when bred to homozygosity, demonstrating that this phenotype was not a property of the transgene itself but could have resulted from a transgene integration disrupting an endogenous gene.

**Fig. S1:**
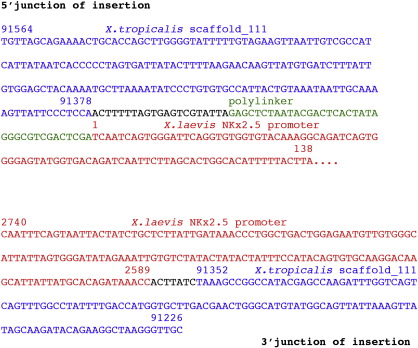
Sequences at either end of the *xdm* insertion were obtained by LM-PCR (5′ junction of insertion with respect to *npnt* transcript) or by PCR with transgene- and scaffold 111-specific primers (3′ end). Sequence corresponding to *X*. *tropicalis* v.4.1 scaffold_111 is shown in blue while sequence of the *X*. *laevis Nkx2.5* promoter is in red; polylinker sequence from the transgene vector is in green; and sequence of unknown origin is in black. Sequences are numbered with respect to the start of scaffold_111 and the *Nkx2.5b* promoter (Genbank accession number: GU573788).

**Fig. 3:**
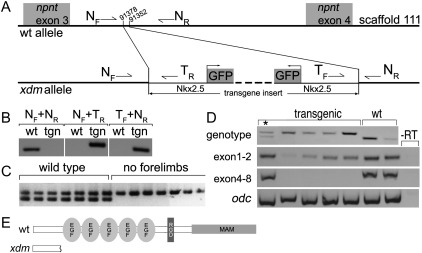
*Xdm* maps to a transgene insertion in *nephronectin*. (A) Schematic showing the wild type *npnt* genomic locus (above), and the structure of the *Nkx2.5*–GFP transgene insertion in the *xdm* allele (below). Genotyping primers which amplify the wild type *npnt* allele (N_F_ + N_R_) and the junctions at each end of the insertion (N_R_ + T_R_ and T_F_ + N_R_) are indicated. (B) *Npnt* sequence spanning the insertion can be amplified with *npnt* primers (N_F_ + N_R_) from wild type (wt) but not transgenic (tgn) haploid embryos; chimaeric DNA at either end of the insertion can be amplified from transgenic but not wild type haploids using an *npnt* primer with a transgene primer (N_F_ + T_R_ and T_F_ + N_R_). (C) Genotyping wild type and forelimbless siblings from an *xdm* cross with N_F_ + N_R_ and N_F_ + T_R_ shows that the *xdm* phenotype is tightly linked to the transgene insertion; sample of 14 shown (175 total genotyped). (D) *Npnt* mRNA is truncated by the transgene insertion. Top panel, 5 transgenic and 2 non-transgenic st. 40 tadpoles genotyped as in (C). Asterisk (left lane) marks heterozygous transgenic tadpole. RT-PCR (2nd and 3rd panels) shows that *npnt* message upstream of transgene insertion (exons 1–2) appears reduced in homozygous *xdm* tadpoles compared to heterozygous or wild type, while exons downstream of the insertion (exons 4–8) are not detected. See Fig. S1 for sequences across junctions of *npnt* intron and transgene. (E) The truncated protein encoded by the *xdm* allele lacks the 5 EGF-like domains, the RGD/linker, and the MAM domain of *nephronectin*.

To characterize the transgene locus, we used Ligation-Mediated PCR (LM-PCR) with primers specific to both ends of the transgene to locate the integration at bp 91378–91352 in scaffold_111 of the JGI *X*. *tropicalis* genome assembly (http://www.genome.jgi-psf.org/Xentr4/Xentr4.home.html) in the third intron of the *nephronectin* (*npnt*) gene (Fig. 3A). Concatameric transgene insertions often result when *Xenopus* eggs are injected with sperm nuclei incubated with linear DNA constructs (Kroll and Amaya, 1996). Only 26 bp of endogenous *npnt* intron sequence is missing in the mutant allele, consistent with a relatively clean transgene insertion (Figs. 3A and S1).

### *Xdm* is genetically linked to *nephronectin*

To address whether the forelimbless phenotype could be caused by a second site mutation, we confirmed its genetic linkage to the transgene insertion. Genotyping primers from endogenous *npnt* intron 3 sequence (N_F_ and N_R_) flanking the insertion site amplify a PCR product from wild type but not transgenic alleles; these primers in combination with transgene primers T_R_ and T_F_ amplify only from transgenic and not wild type (Figs. 3A and B). We used primers N_F_/N_R_ and N_F_/T_R_ to genotype metamorphosing tadpoles from an *xdm* sibling cross. All non-transgene-bearing tadpoles (39/39) developed normal forelimbs. Among transgenic tadpoles, both marker sets produced amplification products from all morphologically wild type larvae (93/93) consistent with heterozygosity at the insertion locus, while all (43/43) metamorphosing animals without forelimbs were homozygous for the transgenic allele (Fig. 3C). A total of 136/136 individuals genotyped therefore lacked recombination events between the transgene insertion and the mutation. Calculating the limit of linkage as if the next tadpole scored had an intervening crossover, the mutation appears closely linked (< 0.8 cm) to the transgene insertion and behaves as a fully penetrant recessive allele.

Insertion of the transgene concatemer disrupts the endogenous *npnt* message structure, as shown by RT-PCR of stage 40 transgenic *xdm* tadpoles (Fig. 3D). *Npnt* message upstream of the insertion (exons 1–2) is expressed in homozygous transgenics, albeit at lower levels than in heterozygous (asterisk) or wild type siblings, but no expression of *npnt* exons downstream of the insertion site is detected. Loss of the downstream exons deletes ~ 85% of the wild type protein, including the 5 EGF-like repeats, the RGD–linker sequence required for *npnt* binding to α8β1 integrin (Sato et al., 2009), and the MAM domain (Fig. 3E). The remaining small N-terminal fragment contains no conserved protein domains, consistent with the mutant being a strong hypomorph or null allele.

### *Nephronectin* morpholino phenocopies *xdm*

**Fig. 4:**
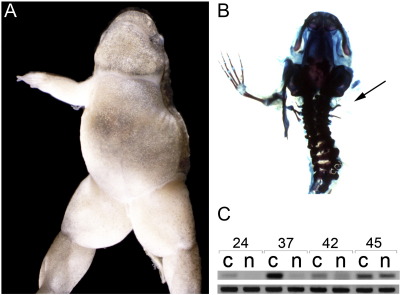
*Npnt* knockdown phenocopies *xdm*. *Npnt* morpholino antisense oligonucleotides were injected into one blastomere of two-cell wild type embryos. (A, ventral view) Injection of two different non-overlapping morpholino sets resulted in unilateral forelimb ablation in a small but statistically significant number of metamorphosing tadpoles. (B) Morpholino knockdown ablating all forelimb skeletal elements, recapitulating the *xdm* phenotype. (C) Upper lanes, RT-PCR of control (c) and *npnt* splice-blocking (n) morpholino injected embryos indicates that *npnt* knockdown is virtually complete at stages 24 and 37, recovering at stage 42, and approaching wt levels at stage 45. Lower lanes, *odc* control.

To confirm a requirement for *npnt* in forelimb formation, we attempted to phenocopy the mutation with antisense morpholino oligonucleotides directed against *npnt* message. Control morpholino injections did not affect forelimb formation (431/431 wild type), but a morpholino targeting the first exon splice donor injected into one cell of two-cell embryos resulted in a statistically significant frequency of one-armed metamorphosing tadpoles (3/98, p < 0.01) which like *xdm* lacked all forelimb skeletal elements on the injected side (Figs. 4A and B). A combination of a different splice-blocking morpholino (directed against the third intron splice donor) and a translation-blocking morpholino also resulted in one-armed metamorphosing larvae (2/47, p < 0.01); neither of these morpholinos injected singly gave significant effects. Injected morpholino oligonucleotides have been shown to be capable of blocking expression until at least stage 43 in *X*. *tropicalis*, with recovery of endogenous gene expression by stage 47 (Nutt et al., 2001). We tried to define a window during which *npnt* was required by evaluating the persistence of the first exon splice-blocking morpholino. RT-PCR using primers spanning exons 1 and 2 shows that correctly-spliced message is depleted by the morpholino at stages 24 and 37, with recovery underway at stage 42 and approaching normal levels by stage 45 (Fig. 4C). Only a small minority (< 4%) of MO-injected embryos display limb defects, so the requirement for *npnt* is likely to occur after splicing has recovered in the majority of embryos. Our results show *npnt* splicing remains blocked at stage 37, consistent with a later requirement for this gene in forelimb initiation. The lower level of *npnt* product in control MO-injected embryos at stages 42 and 45 is consistent with WISH analysis (see Fig. 6). Interpretation of this result is complicated by the low penetrance of the morpholino in blocking forelimb formation. Even at relatively low penetrance, it is striking that *npnt* morpholino injection at cleavage stages can specifically affect limb formation, as forelimb buds first appear in *X*. *tropicalis* 2–3 weeks post-fertilization.

### *Nephronectin* acts upstream of *tbx5* in forelimb bud initiation

**Fig. 5:**
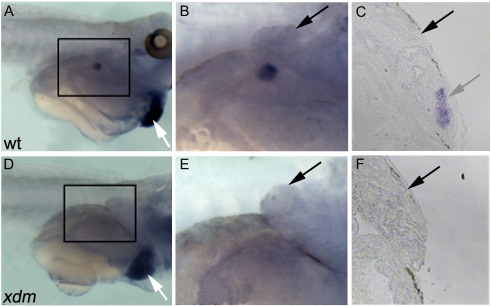
*Xdm* mutants fail to express *tbx5* in the forelimb-forming region. (A–C) WISH analysis of stage 48 wild type embryos detects *tbx5* in the nascent forelimb bud and heart (white arrow). (D–F) *Xdm* embryos lack *tbx5* expression in equivalent flank region. (B, E) Magnified view of boxed regions in (A) and (D) showing pronephros (black arrow). (C) 10× view of a transverse section through wild type flank showing *tbx5* expression in the nascent forelimb (gray arrow) below the pronephros (black arrow). (F) 10× view of a transverse section of *xdm* at equivalent level of the forelimb region shows pronephros (black arrow) but no *tbx5* expression.

In all vertebrate models studied from fish to mice, *Tbx5* is required at the earliest stages of forelimb development for initiation of the forelimb bud (Agarwal et al., 2003; Ahn et al., 2002; Garrity et al., 2002; Ng et al., 2002; Rallis et al., 2003; Takeuchi et al., 2003) as well as for heart development (Brown et al., 2005b; Horb and Thomsen, 1999). Whole mount *in situ* hybridization (WISH) detected *tbx5* staining in the nascent forelimb bud region in wild type (Figs. 5B and C) but not *xdm* (Figs. 5E and F) larvae, while expression in the heart was apparently unaffected (Figs. 5A and D), placing a specific requirement for *npnt* in forelimb initiation upstream of *tbx5* (Ng et al., 2002).

**Fig. 6:**
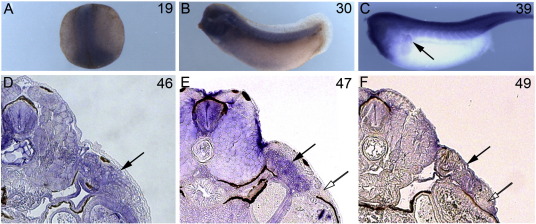
*Npnt* is widely expressed during development. (A) *Npnt* WISH shows strong expression in neural tissue and axial mesoderm in the late neurula (stage 19). (B) Tailbud (stage 30) and tadpole (C, stage 39) express in the head/gill arches, CNS, somites, and pronephros (arrow). (D–F) Transverse sections of *npnt* WISH at stages 46–49 show broad staining including pronephros (black arrow) but exclusion from the epidermis and the prospective forelimb-forming region (open arrow).

**Fig. 7:**
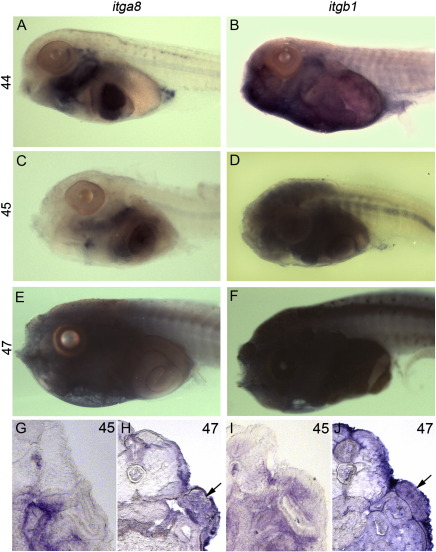
Integrin α8β1 receptor expression at limb forming stages. (A–F) WISH of wild type tadpoles at stages 44, 45, and 47 with probes for the α8 (*itga8*, A, C, E) and β1 (*itgb1*, B, D, F) receptor subunits. (G, H) Transverse sections of α8 and (I, J) β1 WISH at stages 45 and 47. β1 expression is virtually ubiquitous. α8 integrin shows more regionalized expression at stages 44 and 45 in the liver, head cartilage and other structures before becoming broadly expressed by stage 47.

*Npnt* itself is broadly expressed during development. At late neurula (stage 19, Fig. 6A) WISH analysis shows expression in the CNS and axial mesoderm, with additional staining in gill arches at the tailbud stage (stage 30, Fig. 6B). In the swimming tadpole (stage 39, Fig. 6C), *npnt* mRNA is detected in the pronephros adjacent to the forelimb-forming region as well as in the head, somites and CNS. Expression at later stages becomes broad and diffuse, with staining continuing in pronephros during limb initiation (stages 46–49, Figs. 6D–F, black arrows), but apparently absent from the forelimb-forming region (open arrows) and epidermis. We were unable to evaluate protein distribution since available antibodies raised to mammalian *npnt* failed to recognize the *Xenopus* protein. We then analyzed the distribution of *npnt*'s known receptor subunits, α8 and β1 integrins. In *X*. *laevis*, β1 integrin is widely expressed in most tissues (Ransom et al., 1993); WISH analysis in *X*. *tropicalis* confirmed that β1 expression continues to be virtually ubiquitous leading up to forelimb bud formation in both whole mount and section (Figs. 7B, D, F, I, and J). Integrin α8 expression has not been previously described in *Xenopus*, but appears to be more localized than β1 in *X*. *tropicalis*. WISH at stages 44 and 45 (Figs. 7A, C, and G) shows strong expression in the liver and head cartilage, with smaller domains of discrete staining scattered in regions of little or no expression. By stage 47, α8 expression is widespread (Figs. 7E and H). The broad and diffuse expression of both the *npnt* ligand and its α8β1 receptor contrasts sharply with the apparent specificity of the *xdm* phenotype, and does not suggest an instructive role for integrin signalling in induction of forelimb formation. However, the tissue and the developmental window requiring *npnt* function remain to be defined.

**Fig. 8:**
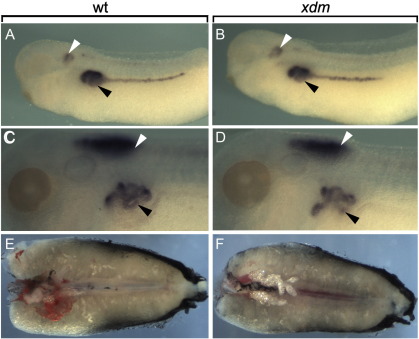
*Npnt* is not required for pronephros or mesonephros formation. (A, B) Specification of pronephros occurs normally as shown by *pax8* WISH in both wt (left panels) and *xdm* (right panels) at the tailbud stage (black arrowheads; white arrowheads show the otic vesicle). (C, D) At stage 40, *xdm pax8* expression in pronephros (black arrowheads) is indistinguishable from wild type (white arrowheads mark hindbrain expression). (E, F) Dissected mesonephros from metamorphosing mutant tadpoles (F) is morphologically indistinguishable from wild type (E); the anterior is to the left.

In mouse, deletion of *npnt* leads to ablation of the metanephros, a kidney organ not found in amphibians. *Xenopus* pronephros and mesonephros were examined to determine whether the mutation affected these amphibian nephric structures. The mutant pronephros was indistinguishable from wild type in its gross morphology, appearance in sections (Figs. 5C and F), and *pax8* expression by WISH at stages 26 and 40 (Figs. 8A–D), and the adult renal organ, the mesonephros, was also morphologically wild type (Fig. 8). Kidney function appears to be normal in *xdm* homozygotes, as no localized edema is seen, and animals are capable of surviving for at least a month post-metamorphosis.

## Discussion

The mechanisms governing limb formation in anurans have not been studied in depth. Here we show that in the *X*. *tropicalis* insertional mutation *xdm*, tadpoles fail to form forelimbs, and that this phenotype maps to a disruption of the *npnt* gene. The defect can be phenocopied by morpholino knockdown of *npnt*, confirming a specific requirement for this gene in forelimb development. Expression of *tbx5*, the earliest known marker of forelimb-forming mesoderm in vertebrates, is ablated in this region in *xdm* embryos, although its expression in the heart is unaffected. Furthermore, all forelimb appendicular skeletal elements are absent, consistent with complete ablation of the entire forelimb program. In mouse, an equivalent loss of all forelimb elements is observed following the conditional limb knockout of *Tbx5* (Rallis et al., 2003), while genetic deletion of *Fgf10*, the apparent direct downstream target of *Tbx5*, produces a slightly milder phenotype with some proximal limb rudiments being formed (Min et al., 1998; Sekine et al., 1999). Together, these data are consistent with *npnt* acting upstream of *tbx5* in frog and implicate a previously unsuspected role for integrin signalling in forelimb initiation. Hindlimb development is entirely unaffected, indicating that the mechanisms that trigger initiation of the hindlimb and forelimbs are different in *X*. *tropicalis*. No other developmental defects have yet been detected in the mutant.

*Npnt'*s role in forelimb formation does not appear to be conserved in mammals, since forelimbs form apparently normally in the mouse knockout (Linton et al., 2007). The mouse mutant, however, shows ablation of the metanephros (a kidney component not present in amphibians), mirroring the kidney phenotype observed in deletions of the α8 integrin subunit of the *npnt* receptor (Muller et al., 1997). A role for nephrogenic mesoderm in forelimb outgrowth was originally suggested by extirpation studies in chick that disrupted wing formation (Geduspan and Solursh, 1992), and gained support with the demonstration that Fgf applied to the flank could induce ectopic limb bud outgrowth (Cohn et al., 1995) and that the developing mesonephros expresses Fgf8 (Crossley and Martin, 1995). This model was recently challenged by reports that forelimb initiation is normal in the absence of the mesonephros (Fernandez-Teran et al., 1997) and that conditional deletion of Fgf8 in mouse mesonephros has no effect on forelimb bud initiation (Perantoni et al., 2005). Pronephric abnormalities also coincide with loss or reduction of the forelimb in the direct-developing frog *Eleutherodactylus coqui* (Lee and Elinson, 2008). Both amphibian kidney components, the adult mesonephros and the larval pronephros, appear unaffected by the *xdm* mutation, and mutants are viable for at least a month after metamorphosis, consistent with wild type renal function. Intriguingly, the prospective forelimb is situated directly adjacent to the tadpole pronephros, where at stage 39 a domain of *npnt* expression is observed, consistent with a role in signalling. At the stages immediately preceding forelimb development, expression of *npnt* becomes diffuse, but persists in the pronephros. Integrin receptors likewise continue to be expressed broadly.

The physiological functions of *npnt* remain poorly understood. It is thought to play a role in transmitting signals from the epithelium to the mesenchyme across the basement membrane through α8β1 integrin (Brandenberger et al., 2001), which it binds with ~ 100× higher affinity than other known ligands such as fibronectin and vitronectin (Sato et al., 2009). *Npnt* can regulate cell migration both in mammalian kidney development and in cancer metastasis (Kuphal et al., 2008; Linton et al., 2007). In zebrafish, pectoral fin precursors migrate in a *Tbx5*-dependent fashion from the lateral plate mesoderm to the fin-forming region (Ahn et al., 2002), and classical microscopy studies describe prospective amphibian hindlimb cells migrating from the coelomic wall to the overlying epidermis (Balinsky, 1931; Tschernoff, 1907). *Xdm* shows a remarkably specific phenotype in limb subtype initiation upstream of *tbx5*, the earliest known marker of the forelimb in vertebrates. While we have been unable to detect localized expression of either ligand or receptor, the population of cells contributing to the initial limb bud formation is likely to be very small and easily missed. Localized post-transcriptional regulation contributing to an instructive role in limb formation also remains a formal possibility. Our data are broadly consistent with *npnt*, possibly secreted by the pronephros, acting as a permissive factor mediating migration of forelimb progenitor cells between body wall and epidermis. Rescue experiments using the *xdm* mutant may help define the cell lineage of the prospective forelimb and the critical window for *npnt* function. It remains to be determined whether the requirement for *npnt* in *Xenopus* forelimb formation is a novel adaptation of the integrin signalling cascade in metamorphosis, or uncovers a conserved mechanism contributing to but not absolutely necessary for vertebrate limb development.

The diploid species *X*. *tropicalis*, with one of the smallest tetrapod genomes, can be used to combine genomic and genetic approaches with *Xenopus*' traditional strengths in embryology, gain-of-function assays, and more recently, transgenesis. Forward genetic screens in vertebrates have been hampered by time-consuming positional cloning, but insertional mutagenesis with known sequence constructs offers alternative mapping strategies (Amsterdam et al., 1999; Bronchain et al., 1999; Hamlet et al., 2006; Yergeau et al., 2007). A key question is the degree to which genetic analysis of this model will be redundant with ongoing studies of zebrafish. The novel role for integrin signalling in forelimb initiation demonstrated by *xdm* shows that at least for tetrapod-specific developmental processes such as limb and lung formation, genetic screens in *X*. *tropicalis* are likely to uncover additional important gene functions.

## Experimental procedures

### Transgenesis

Transgenesis was mediated by nuclear transfer (Amaya and Kroll, 1999; Kroll and Amaya, 1996) using modifications for *X*. *tropicalis* (Hirsch et al., 2002) to integrate a construct containing ~ 7.3 kb of *X*. *laevis* genomic DNA 5′ to the transcriptional start of the *Nkx2*.*5* (GenBank accession: GU573788) gene fused to GFP. Transgenic tadpoles were identified by cardiac fluorescence, grown to adulthood, and outcrossed to wild type; the *xdm* phenotype was observed in progeny of subsequent sibling crosses of one of the transgenic lines.

### Haploid embryos

Haploid embryos were generated by *in vitro* fertilization of eggs from *xdm* carrier females with sperm suspensions irradiated in a Stratalinker at an energy setting of 50,000 μJ.

### Identification of disrupted locus

Sequences flanking one end of the transgene insertion were obtained by LM-PCR with genomic DNA of homozygous *xdm* tadpoles using the GenomeWalker Universal Kit (Clonetech, 638904) and BD Advantage™ 2 polymerase mix (Clonetech, 639201), according to the manufacturer's instructions. The transgene-specific primer, designed from the *Nkx2.5* promoter sequence was: GACTTCAGCTCTTTGGAAATTCGGAAC.

Following identification of one end of the integrated locus, the other border was amplified for sequencing with transgene and *npnt* intron 3 specific primers with the following sequence:

F-CGTCCGTCCGTCTTTCTATC
R-GGGTCATTTGAAGACACCAGA.

### RT-PCR

RNA was prepared using Trizol (Invitrogen). cDNA was prepared and amplified with the Enhanced Avian HS RT-PCR kit (Sigma HSRT-100) using the following primers:

exons 1–2

F-TGCTGTGCTTCTCCCTATCA
R-CATCCCCAGCAGCAGTCTAT

exons 4–8

F-AACATGGGGACTGTGTAGGC
R-GATACTGGCCCGTGTAGCAT

*odc*

F-GCCAGTAAGACGGAAATCCA
R-CCCATGTCAAAGACACATCG.

### Staging

Embryos and tadpoles were staged according to Nieuwkoop and Faber, with precedence at stages > 45 given to gut coiling and limb morphology criteria, as pigmentation differs slightly in *X*. *tropicalis*.

### Whole mount *in situ* hybridization

Fixation and WISH were carried out as described previously (Sive et al., 2000) with modification of protease treatment to 15 mg/ml Proteinase K for 30′ at 37 °C. WISH probes were obtained by subcloning RT-PCR products into the PCRII-TOPO vector using the TOPO TA Cloning Kit (Invitrogen, K4600-40) and confirmed by sequencing, except the *itgb1* WISH probe transcribed from IMAGE clone 6995562. Primers used were:

*npnt*

F-AACATGGGGACTGTGTAGGC
R-GATACTGGCCCGTGTAGCAT

*tbx-5*

F-GACACATCGCCAAGTGAAGA
R-ACAAGAATTCCGTGGGTTGA

*itga8*

F-CCCAAAGCCAATACCTCTCA
R-AACGCCAGGTCTCCATTCTT.

### Genotyping

Primers used to genotype embryos were as follows:

N_F_-ATCCCCTGTGTGCCATTACT
N_R_ GGGTCATTTGAAGACACCAGA
T_F_ CACCTGCCTTGCTAAAAGGA
T_R_-GGAGATTCGGGACTTAACAGG

### Morpholino injections

Morpholinos were purchased from GeneTools LLC. A total of 12 ng of morpholino was injected into one cell of a two-cell embryo.

Morpholino sequences were as follows:

*npnt* translation-blocking morpholino: TATTAACCTGATTGCTAACTCCATG
*npnt* morpholino blocking 1st coding exon splice donor: ATTCCAGCCATTCTTACCTTCCATC
*npnt* morpholino blocking 4th coding exon splice donor: TGGATATAATCTATCCATACCTTGG

Control morpholino:

CCTCTTACCTCAGTTACAATTTATA

### Statistical analysis

P values were calculated from 2 × 2 contingency tables using Fisher's exact test.

The following is the supplementary material related to this article.
